# Physiology, Biochemistry and Pharmacology of Transporters for Organic Cations 2.0

**DOI:** 10.3390/ijms23116328

**Published:** 2022-06-06

**Authors:** Giuliano Ciarimboli

**Affiliations:** Experimental Nephrology, Department of Internal Medicine D, University Hospital Münster, 48149 Münster, Germany; gciari@uni-muenster.de

This editorial summarizes the 12 scientific papers published in the Special Issue “Physiology, Biochemistry, and Pharmacology of Transporters for Organic Cations 2.0” of the *International Journal of Molecular Sciences*. In this Special Issue, the readers will find integrative information on transporters for organic cations. Many of these transporters belong to the solute carrier (SLC) families 22 (SLC22) and 47 (SLC47). In addition to a review on the physiology, pharmacology, and toxicology of the Novel Organic Cation Transporter, OCTN1 [1], which offers a concise overview of the field, the readers will find 11 original research works focusing on specific aspects of transporter physiology and pharmacology.

Specifically, the review “OCTN1: A Widely Studied but Still Enigmatic Organic Cation Transporter Linked to Human Pathology and Drug Interactions” by Pochini et al. [1] summarizes the most recent advances in the research on OCTN1 function, physiological roles, substrate specificity, drug interactions, tissue expression, and relationships with pathology.

In the paper “SLC22 Transporters in the Fly Renal System Regulate Response to Oxidative Stress In Vivo”, Zhang et al. [2] used tissue-selective knockdown strategies of four Drosophila *SLC22* genes (*CG6126*, *CG16727*, *CG6006*, and *CG4630*) to investigate their role in oxidative stress induced by paraquat in flies. This study demonstrates that, when compared to control lines, decreased transporter expression resulted in significantly increased resistance to oxidative stress. Therefore, the authors suggest that SLC22 transporters within the fly renal system and their human relatives may probably have a similar role in oxidative stress and organ crosstalk.

The paper “Cloning and Functional Characterization of Dog OCT1 and OCT2: Another Step in Exploring Species Differences in Organic Cation Transporters” by Meyer at al. [3] is another important comparative study on the species-specific properties of organic cation transporters (OCTs). Analyzing the sequences of dog organic cation transporter 1 (dog OCT1) and dog OCT2, they found that the correct dog OCT1 sequence differs from the annotated dog genome assembly in the NCBI database (CanFam3.1). In this work, it was found that experimentally determined sequences of dog OCT1 and OCT2 share 80% and 81% amino acid identity with human OCT1 (hOCT1) and OCT2, respectively. Dog OCT1 was highly expressed in both the liver and the kidney, while dog OCT2 was highly expressed in the kidney, but not in the liver. After expression in human embryonic kidney (HEK) cells, the transport properties of dog and human OCT1 and 2 were compared. Dog OCT1 transported fenoterol with higher capacity but lower affinity than hOCT1. Conversely, hOCT1 transported ipratropium with higher capacity but lower affinity than dog OCT1. Compared to hOCT2, dog OCT2 showed the lower transport of fenoterol and butylscopolamine. These results are important when using dogs as pre-clinical models and also for dog drug therapy.

When studying whether a substance interacts with a transporter, the inhibition of transport of a tracer substrate by the substance under investigation (*cis*-inhibition) is often used. However, this approach is, in many cases, not able to discriminate between transported and non-transported inhibitors. This aspect is often investigated using *trans*-stimulation assays. Therefore, in the work “Substrate-Dependent Trans-Stimulation of Organic Cation Transporter 2 Activity”, Lefèvre et al. [4] evaluated the *cis*-inhibition and *trans*-stimulation properties of 15 hOCT2 substrates on the transport of the fluorescent OCT2 tracer 4-(4-(dimethylamino)styryl)-N-methylpyridinium (DiASP) in HEK cells overexpressing hOCT2. They found that all tested substrates *cis*-inhibited DiASP uptake by hOCT2, but only four substrates (acetylcholine, agmatine, choline, and metformin) were able to *trans*-stimulate the transporter. The authors concluded that the *trans*-stimulation assay has a rather low sensitivity for identifying OCT2 substrates, and caution with respect to the use of such assay should therefore be considered.

In epithelial tissues, organic cation transporters show a polar distribution, being mainly localized in the basolateral plasma membrane domain. Therefore, Koepp et al. [5] in their work “Properties of Transport Mediated by the Human Organic Cation Transporter 2 Studied in a Polarized Three-Dimensional Epithelial Cell Culture Model” stably expressed hOCT2 in Madin-Darby Canine Kidney (MDCK) cells and cultivated these cells in the presence of an extracellular matrix to obtain three-dimensional (3D) structures (cysts). In this system, hOCT2 showed a specific localization in the basolateral membrane domain. Transport properties of hOCT2 expressed in MDCK cysts were compared with those measured using HEK cells stably transfected with hOCT2. The results of this study suggest that expression in a 3D structure may change the apparent affinity of hOCT2 for some substances and also its acute regulation by signaling pathways, which are active in polarized cells.

The renal secretion of organic cations in proximal tubules is a vectorial process requiring the cellular uptake of organic cations at the basolateral membrane domain, which is mainly mediated by hOCT2, and their extrusion into the urine, which is mainly mediated by the human multidrug and toxin extruder 1 (hMATE1). Therefore, determination of the transporter turnover rate (TOR) is relevant to interpret the in vitro–in vivo extrapolation (IVIVE) required for the physiologically based pharmacokinetic (PBPK) modelling of organic cation renal secretion. Zhang and Wright, in their work, “Transport Turnover Rates for Human OCT2 and MATE1 Expressed in Chinese Hamster Ovary-CHO-Cells” [6], determined TORs for hMATE1 and hOCT2 proteins expressed in CHO cells. To calculate TORs, two parameters are required: the number of functional transporters expressed by a particular cell or tissue and the maximal rate of transport mediated by each transporter. The authors calculated for hMATE1 TOR values of 297 and 1894 s^−1^ for 1-methyl-4-phenylpyridinium (MPP^+^) and metformin, respectively. For hOCT2, the TOR values were 8.0 and 70 s^−1^ for MPP^+^ and metformin, respectively. These TOR values are consistent with what has been found for a variety of other transporters.

OCTs are polyspecific transporters, meaning that they can accept several different substances as substrate. Because of this behavior, they can interact with many different drugs and have potential pharmacological implications. Therefore, a screening method for inhibitors of OCTs without employing labelled substrates will greatly simplify the determination of possible interactions of drugs with OCTs. In the paper “MPP^+^-Induced Changes in Cellular Impedance as a Measure for Organic Cation Transporter (SLC22A1-3) Activity and Inhibition”, Mocking et al. [7] established a label-free impedance-based transport assay able to detect OCT-mediated transport activity and inhibition utilizing the neurotoxin MPP^+^. The uptake of MPP^+^ by OCTs induced concentration-dependent changes in cellular impedance that were inhibited by decynium-22, corticosterone, and tyrosine kinase inhibitors.

Focusing further on the pharmacological implications of transporter-mediated drug uptake, in the paper “Expression and Functional Contribution of Different Organic Cation Transporters to the Cellular Uptake of Doxorubicin into Human Breast Cancer and Cardiac Tissue”, Otter et al. [8] investigated the role of plasma membrane transporters for the effects and side effects (cardiotoxicity) of the anticancer drug doxorubicin. Doxorubicin could be identified as a substrate of OCT1, OCT2, OCT3, and of organic anion transporting polypeptide 1A2 (OATP1A2). These transporters are expressed in breast cancer tissues and/or the human heart and may be involved in the determination of effects and side effects of doxorubicin. Transporters’ genetic variants and the excipient Cremophor EL can decrease the activity of the transporters.

Platinum derivatives such as Cisplatin and Oxaliplatin are other anticancer drugs that are substrates of hOCT2. The interaction of these Platinum derivatives with hOCT2 seems to be responsible for the development of their side-effects (nephrotoxicity for Cisplatin and peripheral neurotoxicity for Oxaliplatin) but not for their chemotherapeutic action. Therefore, much research work is aimed to find a way to reduce these side-effects of Platinum derivatives. Autophagy seems to be a mechanism by which cells can protect themselves against damage induced by pharmacological treatment. In the paper “Role of Organic Cation Transporter 2 in Autophagy Induced by Platinum Derivatives”, Ahmed-Eltayeb et al. [9] investigated the role of hOCT2 on autophagy induced by Cisplatin and Oxaliplatin. They also explored the effect of autophagy activation by starvation on the toxicities of these Platinum derivatives. The results of this study indicate that autophagy is induced in response to Cisplatin and Oxaliplatin in HEK cells overexpressing hOCT2 but not in wild-type HEK cells. Furthermore, the inhibition of autophagy is associated with the higher toxicity of Platinum derivatives, and starvation was found to offer protection against Cisplatin-associated toxicity. In conclusion, the activation of autophagy could be a potential strategy to protect against unwanted toxicities induced by treatment with Platinum derivatives.

Transporters also have an important role in the central nervous system. For example, the abuse of amphetamine-like psychostimulants leads to a variety of adverse effects, which are probably mediated by their interaction with transporters. Amphetamine is a substrate for the high-affinity dopamine (DA), norepinephrine (NE), and serotonin (5-HT) transporters (DAT, NET, and SERT, respectively). However, strategies targeting DAT, or SERT and NET, to treat amphetamine addiction have failed. In the paper “Role of Organic Cation Transporter 3 and Plasma Membrane Monoamine Transporter in the Rewarding Properties and Locomotor Sensitizing Effects of Amphetamine in Male and Female Mice”, Clauss et al. [10] showed that both OCT3 and the plasma membrane monoamine transporter (PMAT) appear to be important for the development of sensitization to the locomotor stimulant effect of amphetamine in female and PMAT in male mice. These findings support an important, sex-dependent role of OCT3 and PMAT in the rewarding and locomotor stimulant effects of amphetamine, suggesting that OCT3 and PMAT may be novel targets for the development of treatments for addiction to amphetamine and related psychostimulants.

Still focusing on human OCT subtypes and hPMAT, the paper “Interaction Profiles of Central Nervous System Active Drugs at Human Organic Cation Transporters 1–3 and Human Plasma Membrane Monoamine Transporter” by Angenoorth et al. [11] investigates the interaction of 17 psychoactive substances with these transporters utilizing a radiotracer-based in vitro uptake inhibition assays in HEK cells overexpressing hOCTs or hPMAT. They showed that many compounds inhibit substrate uptake by hOCT1 and hOCT2 in the low micromolar range, whereas only a few substances interact with hOCT3 and hPMAT. Interestingly, methylphenidate and ketamine selectively interact with hOCT1 or hOCT2, respectively. Additionally, 3,4-methylenedioxymethamphetamine (MDMA) is a potent inhibitor of hOCT1 and 2 and hPMAT. Enantiospecific differences of R- and S-α-pyrrolidinovalerophenone (R- and S-α-PVP) and R- and S-citalopram were also explored. No distinct enantioselective differences in uptake inhibition between the two compounds on any transporter was observed.

Using HEK cells overexpressing human monoamine transporters (MATs) or OCTs, in the paper “Overlap and Specificity in the Substrate Spectra of Human Monoamine Transporters and Organic Cation Transporters 1, 2, and 3”, Gebauer et al. [12] compared the uptake of 48 compounds, mainly phenethylamine and tryptamine derivatives, including matched molecular pairs, across noradrenaline, dopamine, and serotonin transporters and OCTs (1–3). They found that MATs showed high transport activities for numerous analogues of neurotransmitters, but their substrate spectra were limited by molar mass. Human OCT2 showed the broadest substrate spectrum and the highest overlap with MATs substrates. The radiotracer meta-iodobenzylguanidine showed the most balanced uptake across all six transporters. These data are important for a better understanding of pharmacokinetics and toxicokinetics of small molecular organic cations.
